# Antibiofilm activity and NMR-based metabolomic characterization of cell-free supernatant of *Limosilactobacillus reuteri* DSM 17938

**DOI:** 10.3389/fmicb.2023.1128275

**Published:** 2023-02-20

**Authors:** Irene Vitale, Mattia Spano, Valentina Puca, Simone Carradori, Stefania Cesa, Beatrice Marinacci, Francesca Sisto, Stefan Roos, Gianfranco Grompone, Rossella Grande

**Affiliations:** ^1^Department of Pharmacy, “G. d’Annunzio” University of Chieti-Pescara, Chieti, Italy; ^2^Department of Drug Chemistry and Technology, Sapienza University of Rome, Rome, Italy; ^3^Department of Innovative Technologies in Medicine & Dentistry, “G. d’Annunzio” University of Chieti-Pescara, Chieti, Italy; ^4^Department of Biomedical, Surgical and Dental Sciences, University of Milan, Milan, Italy; ^5^Department of Molecular Sciences, Swedish University of Agricultural Sciences, Uppsala, Sweden; ^6^BioGaia AB, Stockholm, Sweden; ^7^Center for Advanced Studies and Technology (CAST), G. d’Annunzio University of Chieti-Pescara, Chieti, Italy

**Keywords:** *Lactobacillus reuteri*, *Limosilactobacillus reuteri*, cell-free supernatant, NMR, color analysis, antibiofilm activity, probiotics, postbiotics

## Abstract

The microbial biofilm has been defined as a “key virulence factor” for a multitude of microorganisms associated with chronic infections. Its multifactorial nature and variability, as well as an increase in antimicrobial resistance, suggest the need to identify new compounds as alternatives to the commonly used antimicrobials. The aim of this study was to assess the antibiofilm activity of cell-free supernatant (CFS) and its sub-fractions (SurE 10 K with a molecular weight <10 kDa and SurE with a molecular weight <30 kDa), produced by *Limosilactobacillus reuteri* DSM 17938, vs. biofilm-producing bacterial species. The minimum inhibitory biofilm concentration (MBIC) and the minimum biofilm eradication concentration (MBEC) were determined *via* three different methods and an NMR metabolomic analysis of CFS and SurE 10K was performed to identify and quantify several compounds. Finally, the storage stability of these postbiotics was evaluated by a colorimetric assay by analyzing changes in the CIEL*a*b parameters. The CFS showed a promising antibiofilm activity against the biofilm developed by clinically relevant microorganisms. The NMR of CFS and SurE 10K identifies and quantifies several compounds, mainly organic acids and amino acids, with lactate being the most abundant metabolite in all the analyzed samples. The CFS and SurE 10 K were characterized by a similar qualitative profile, with the exception of formate and glycine detected only in the CFS. Finally, the CIEL*a*b parameters assess the better conditions to analyze and use these matrices for the correct preservation of bioactive compounds.

**Figure:**
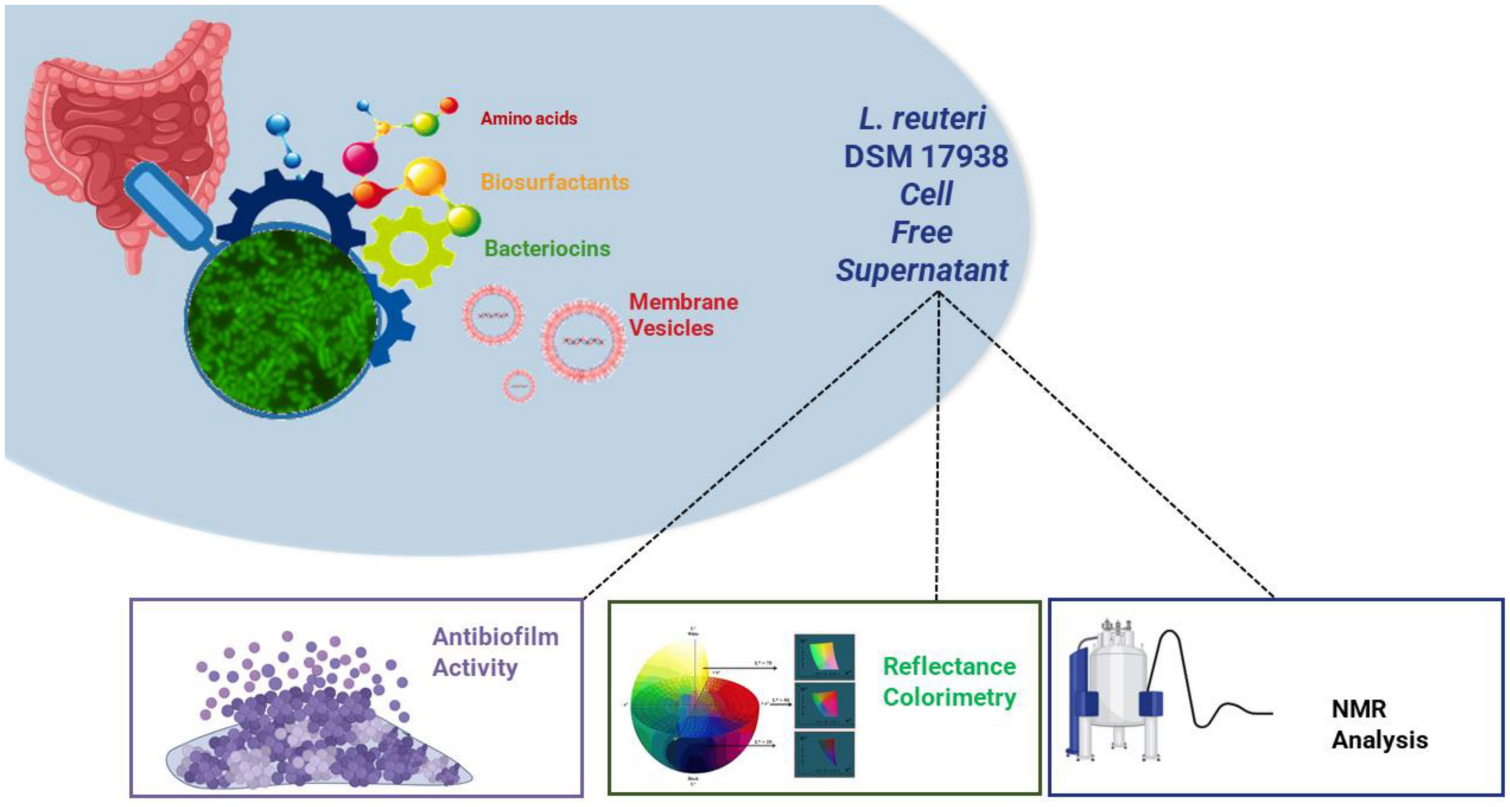
GRAPHICAL ABSTRACT.

## Introduction

The microbial biofilm represents a microbial strategy of cooperation in which the microorganisms aggregate, communicate, and defend themselves thanks to the extracellular polymeric substances (EPS) that constitute a protective barrier (Hall-Stoodley et al., 2004; Flemming et al., 2016). It has always been believed that the formation of biofilm began following the adhesion of “pioneer microorganisms” on a biotic or abiotic surface; therefore, biofilms are defined as “manifestations of microbial life, not only growing on surfaces, but developing at any solid–liquid, liquid–liquid, liquid–gas, and solid–gas interfaces” (Flemming et al., 2021). The EPS matrix is very heterogeneous and complex and depends on many environmental factors as well as on bacterial strains, culture conditions, and biofilm age (Allesen-Holm et al., 2006; Grande et al., 2014). It can be constituted from polysaccharides, proteins, lipids, insoluble components such as amyloids, fimbriae, pili, flagella, and nucleic acids, in particular extracellular DNA (eDNA), released as free components or associated with extracellular vesicles (Hall-Stoodley et al., 2004; Grande et al., 2015). Regarding the eDNA, we showed in our previous work that it can play a different role in membrane vesicles, depending on the bacterial phenotype (Grande et al., 2015, 2017).

Koo et al. (2017) recognized microbial biofilm formation as a “key virulence factor” for numerous microorganisms responsible for chronic infections. The tolerance expressed by biofilms against traditional antimicrobial drugs as well as the host immune defenses makes them difficult to eradicate, contributing to the spread of the antibiotic resistance phenomenon (Grande et al., 2021). A wide range of microorganisms that possess different characteristics (Gram-positive and Gram-negative, motile and non-motile, aerobic, anerobic, and microaerophilic) are biofilm producers, and many of them are opportunistic pathogens associated with hospital-acquired infections. Based on the consideration that biofilm infections can be derived from dysbiosis, the characterization of natural compounds produced by probiotic strains could represent a new goal to achieve (Grande et al., 2020). The cell-free supernatant (CFS) produced by many probiotic strains (e.g., *Limosilactobacillus reuteri*) contains many bioactive compounds such as biosurfactants, bacteriocins, and antimicrobial peptides, free or delivered by extracellular vesicles, with inhibitory effects on the growth of the pathogen (Alakomi et al., 2000; Aminnezhad et al., 2015; Grande et al., 2017; Maccelli et al., 2020). However, each probiotic strain produces its own compounds. In a previous report, we demonstrated that *Limosilactobacillus reuteri* DSM 17938 CFS has antimicrobial activity vs. both Gram-negative and Gram-positive bacteria, while membrane vesicles of planktonic phenotype (pMVs) and biofilm phenotype (bMVs) were not active (Maccelli et al., 2020). The CFS and its subfraction SurE 10 K, which contains molecules or compounds with a molecular size lower than 10 kDa, were tested at different concentrations against reference and clinical strains of *Escherichia coli*, *Pseudomonas aeruginosa*, *Fusobacterium nucleatum*, *Staphylococcus aureus*, and *Streptococcus mutans* characterized by different antimicrobial susceptibility patterns. The results demonstrated that the CFS contained antimicrobial compounds, in addition to the well-known reuterin, showing greater activity against Gram-negative bacteria than Gram-positive bacteria, and the efficacy was related to the species rather than to the individual strains (Maccelli et al., 2020). Moreover, the data obtained supported the hypothesis that the antimicrobial effect could be associated with synergistic activity between several compounds contained in the CFS, as previously demonstrated by other authors (Poppi et al., 2015).

**Figure 1:**
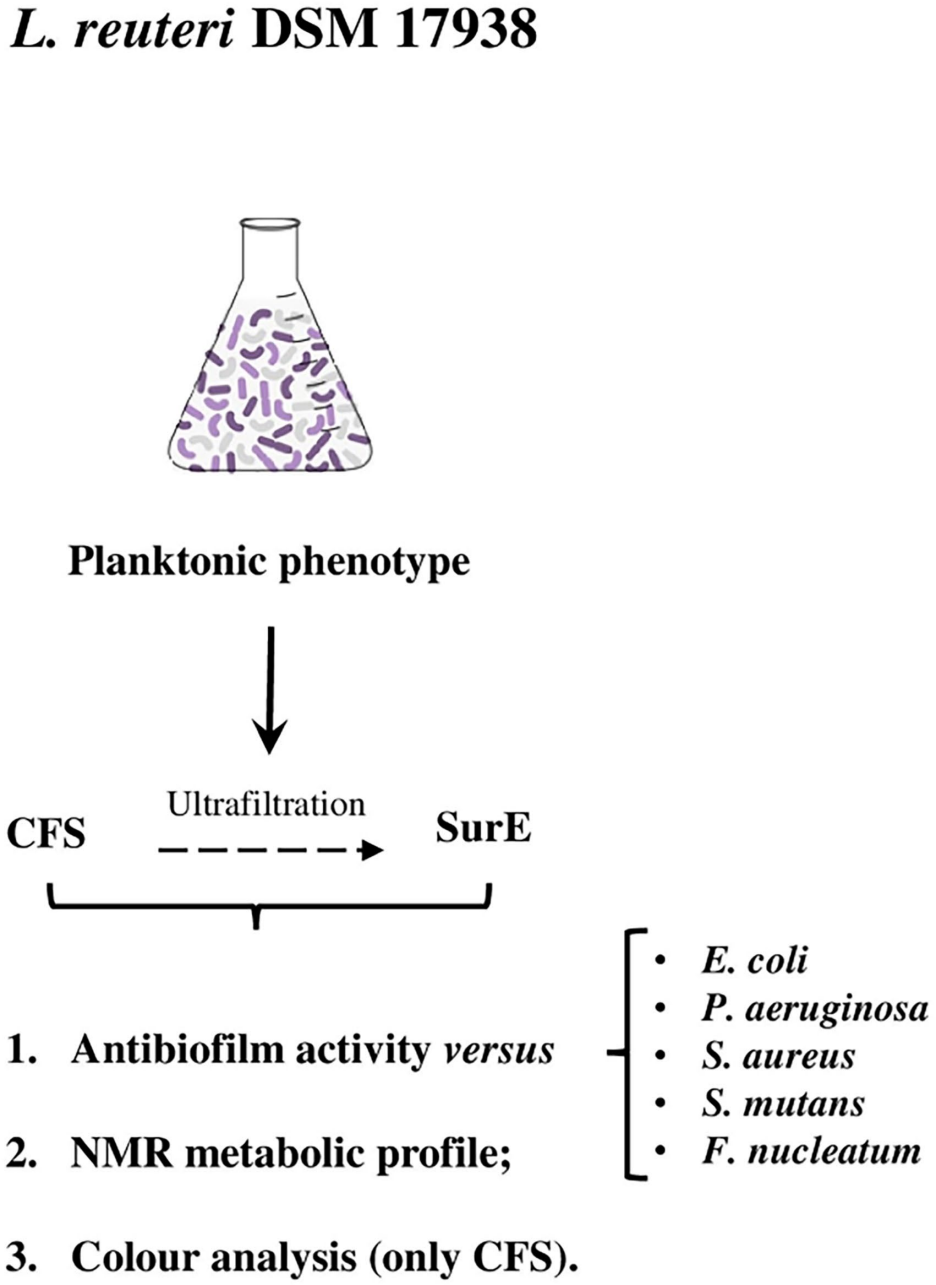
Flowchart of this study. CFS, cell-free supernatant; SurE, eluted supernatant <10 kDa.

On the basis of the results previously obtained and considering the clinical relevance of microbial biofilms, the first aim of this study was to evaluate the antibiofilm activity of the CFS and some of its fractions (SurE 10 K and 30 K). We chose clinically relevant species that form biofilms to test the capability of the CFS to both inhibit biofilm formation and eradicate mature biofilms. Successively, to better comprehend the potential of this probiotic, we aimed at improving our previous mass spectrometry-based knowledge of the metabolic content by means of qualitative and quantitative NMR analyses. Given that the chemical composition of the entire set of metabolites of a probiotic is very complex, an untargeted metabolite profile characterization of this matrix can be useful to identify the main metabolites of the considered system and potentially correlate them with the biological activity. In this context, NMR spectroscopy represents a very powerful tool for this kind of analysis since this methodology can be used to obtain a chemical profile of a biological system (Bianchi et al., 2020). Finally, we monitored the stability of microbial products with an affordable and fast color analysis assay. Previous studies on cyanobacteria monitored biofilm formation and how pigment production was affected by the environment (Sanmartín et al., 2010, 2012). Our previous studies demonstrated how color could be useful to monitor the shelf-life of different systems, providing useful information about oxidative stability and, more generally, about browning, which represents the main modification induced by aging on the biological matrices (Cesa et al., 2015; Menghini et al., 2021). In this view, a preliminary shelf-life stability study to monitor the chemical composition of CFS was performed by CIEL*a*b colorimetric analysis for a period of 4 weeks. The workflow described earlier is shown in Figure 1.

## Materials and methods

### Bacterial strains and culture conditions

*Limosilactobacillus reuteri* DSM 17938 (Rosander et al., 2008), provided by BioGaia AB (Stockholm, Sweden), was used in the study. The bacteria were plated on DeMan, Rogosa, and Sharpe agar (MRSA; Oxoid Limited, Hampshire, United Kingdom), and incubated at 37°C for 24 h in an anaerobic atmosphere (Anaerogen Pak Jar, Oxoid Ltd.). The CFS and its fractions, SurE 10 K and 30 K, were obtained following the procedure described by Maccelli et al. (2020). Regarding the other bacterial strains used, different media and growth conditions were used on the basis of the species.

### Biofilm formation assay

The biofilms were developed on 96-well flat-bottomed polystyrene microtitre plates (Eppendorf, Hamburg, Germany) by using different media and growth conditions depending on the species. The OD_600_ of each inoculum was read and adjusted to reach a final concentration in each well of 1.0 × 10^5^ CFU/mL. More details are given as follows:

- *Escherichia coli* ATCC 25922 was grown in Mueller Hinton Broth (MHB; Oxoid Limited, Hampshire, United Kingdom), and the biofilm was developed in MHB for 24 h of incubation at 37°C in static and aerobic conditions;
- *Pseudomonas aeruginosa* ATCC 27853 growth and biofilm formation were conducted in Luria Bertani Broth (LB; Oxoid Limited, Hampshire, United Kingdom) for 24 h of incubation at 37°C in static and aerobic conditions;
- *Streptococcus mutans* UA 159 was grown in Brain Heart Infusion (BHI; Oxoid Limited, Hampshire, United Kingdom), and the biofilm was developed in BHI + 1% of sucrose (BHIS) for 24 h of incubation at 37°C in static and anaerobic conditions;
- *Staphylococcus aureus* ATCC 29213 was grown in Tryptic Soy Broth (TSB; Oxoid Limited, Hampshire, United Kingdom), and the biofilm was developed in TSB + 1% of glucose (TSBG) for 24 h of incubation at 37°C in static and aerobic conditions;
- *Fusobacterium nucleatum* ATCC 25586 growth and biofilm formation were conducted in BHI for 48 h of incubation at 37°C in static and anaerobic conditions.

### Determination of CFS and SurE 10 K minimum biofilm eradication concentration

The MIC and MBC of the CFS and SurE 10 K vs. *P. aeruginosa* ATCC 27853, *F. nucleatum* ATCC 25586, *E. coli* ATCC 25922, *S. aureus* ATCC 29213, and *S. mutans* UA159 were previously determined (Maccelli et al., 2020).

Regarding the determination of the Minimum Biofilm Eradication Concentration (MBEC), the biofilms were developed depending on the bacterial species. The MBEC was defined as the lowest concentration, which completely eradicated bacterial biofilms developed in 96-well flat-bottomed polystyrene microtiter plates. At the end of incubation, the biofilms were washed with phosphate-buffered saline (PBS), and the CFS and SurE 10 K were added to the pre-formed biofilms at concentrations corresponding to 1 × MIC, 2 × MIC, 3 × MIC, and 4 × MIC of each test sample, except for the *F. nucleatum* biofilm that was treated with 2 × MIC, 3 × MIC, 4 × MIC, and 5 × MIC. Controls consist of (i) non-treated biofilms and (ii) biofilms with the addition of MRS broth (the medium in which CFS and SurE are resuspended). The microtiter plates were then incubated at 37°C for the time of incubation chosen on the basis of bacterial species under static conditions. The eradication effects of CFS and SurE 10 K were measured using three different methods, namely Colony Forming Units (CFU) counting; metabolic assays such as alamarBlue^®^ (AB) (Thermo Fisher Scientific, Waltham, MA, United States) or XTT (sodium 3’-[1-(phenylaminocarbonyl)-3,4-tetrazolium]-bis-(4-methoxy-6-nitro)benzene sulfonic acid hydrate; Cell Proliferation Kit II XTT, Roche Diagnostic, Mannheim, Germany); and Crystal Violet (CV) assay.

### Cell viability evaluation through colony-forming unit count

The CFU enumeration was performed to evaluate the bacterial cell viability in the treated and non-treated biofilms with different concentrations of CFS and SurE 10 K. A volume of 100 μL of the sample was taken from each well and used for CFU counts. Serial dilutions of the stock were performed in PBS (pH 7.2) and plated on the suitable agar media at 37°C for 18–24 h. The same evaluation was performed in the case of the AB assay due to its non-toxic nature.

### Cell viability assays

The MBEC was confirmed using the XTT metabolic assay and/or AB assay according to the manufacturer’s recommendations (Zengin et al., 2018; Maccelli et al., 2020). Given that we noticed that some components or supplements present in the media that were used interfered with the dyes present in the kits, it was decided to use both kits and compare the data obtained.

The absorbances corresponding to 490 nm for XTT and 570 and 600 nm for AB were then read with a microplate reader (Synergy H1 Multi-Mode Reader, BioTek, Winooski, VT, United States). The XTT and Alamar Blue MBEC were defined as the lowest concentrations of the sample, resulting in a detectable colorimetric change in the assays. The colorimetric change is a direct correlation with the metabolic activity of the biofilm. Two independent experiments were performed in triplicate.

### Crystal violet assay

The Crystal Violet assay was used to stain the biofilm biomass. The treated and non-treated biofilms were rinsed with 100 μL of PBS and fixed for 1 h at 60°C. Then, 100 μL of crystal violet at 0.1% diluted in MilliQ water was added for 5 min to *P. aeruginosa*, *S. mutans*, *E. coli*, and *S. aureus* pre-formed biofilms. Crystal violet at 0.5% diluted in MilliQ water was added for 2 min to pre-formed biofilm of *F. nucleatum*. The biofilms were washed with 200 μL of MilliQ water and dried at room temperature (RT) for 30 min. In the end, 100 μL of 33% acid acetic was added to each well for 10 min and absorbance was read at 590 nm.

### Live/dead assay

The qualitative analysis of the CFS-treated and untreated biofilms developed by the different bacterial species was performed using live/dead staining (BacLight kit; Thermo Fisher Scientific, Waltham, MA, United States) as indicated by the manufacturers and fluorescent microscopy evaluation with a Leica DMR Fluorescent Microscope (Leica Microsystem, Wetzlar, Germany). Two experiments were performed in triplicate.

### Determination of SurE 30 K minimum biofilm eradication concentration vs. *Pseudomonas aeruginosa* ATCC 27853

SurE 30 K was obtained *via* ultrafiltration of CFS with Amicon Ultra-15 30 K (30000 MWCO) Centrifugal Filter Devices (Merck KGaA, Darmstadt, Germany). The MIC of SurE 30 K was determined as for SurE 10 K (data not shown; Maccelli et al., 2020). After determining the MIC, a *P. aeruginosa* biofilm was developed in LB, and after 24 h, the mature biofilm was treated with SurE 30 K at 1 × MIC, 2 × MIC, 3 × MIC, and 4 × MIC. The MBEC was evaluated with an AB assay, CFU counting, and CV assay.

### Minimum biofilm inhibitory concentration of CFS and SurE 10 K

To determine the MBIC, the CFS and SurE 10 K were diluted into each well until reaching a concentration of half of the Minimum Inhibitory Concentration (MIC). The MIC values of *L. reuteri* CFS and SurE 10 K vs. the bacterial species used were previously described (Maccelli et al., 2020). Then, the OD_600_ of each inoculum was read and adjusted to reach a final concentration in each well of 1.0 × 10^5^ CFU/mL. The plates were incubated at 37°C for 24 h for *P. aeruginosa*, *S. mutans*, *S. aureus*, and *E. coli* and for 48 h for *F. nucleatum.* The valuation of the MBIC was determined by both CFU counts and metabolic assays previously described.

### Determination of minimum inhibitory concentration of CFS and SurE 10 K vs. *Lactobacillus rhamnosus* ATCC 53103, *Lactobacillus paracasei* CNCM I-1572, and *Lactobacillus acidophilus* LA14 ATCC SD 5212

The minimum inhibitory concentration was determined using the broth microdilution assay in 96-well polystyrene microtiter plates and CFU counting. Briefly, *Lactobacillus rhamnosus*, *Lactobacillus paracasei*, and *Lactobacillus acidophilus* were grown in MRSB for 17 h at 37°C under shaking conditions at 125 rpm. The overnight cultures were resuspended until reaching an optical density of 0.1 nm (OD_600_) that corresponds to 10^7^ CFU/mL and then diluted to 10^5^ CFU/mL in the 96-well plate. CFS and SurE 10 K were tested from a maximum concentration of 88 μL/100 μL to a minimum concentration of 5.5 μL/100 μL. The plates were incubated in static and anaerobic conditions for 24 h at 37°C. The MIC was defined as the lowest concentration without visible growth, and it was determined both visually and *via* CFU counting. Controls consisted of MRSB media without the addition of CFS. Two independent experiments were performed in triplicate.

### Determination of MIC and MBC values of lactic acid vs. *Staphylococcus aureus*

The antimicrobial activity of lactic acid (Puraq Bioquimica SRL, Spain) was tested vs. *Staphylococcus aureus* ATCC 29213 *via* the broth microdilution method in 96-well plates according to the CLSI guidelines and confirmed by XTT assay, following the methods previously described (Maccelli et al., 2020). Lactic acid was diluted in MHB and tested in the range of 0.05–6.4 μL/100 μL.

### Statistical analysis

The differences in the means of the results between untreated and treated bacterial strains were evaluated by one-way ANOVA (GraphPad Software, San Diego, CA, United States) and Dunnet’s multiple comparison test. Media supplemented with MRS broth at the maximum concentration of CFS tested was used as the control. The probability value of *p* ≤ 0.05 was considered significantly different. Analysis of cytotoxicity data (expressed as mean ± standard error) was performed by the GraphPad Prism TM 6.00 software (GraphPad Software, San Diego, CA, United States).

### NMR-based qualitative and quantitative analyses

A volume of 1 mL of each sample was lyophilized and then dissolved in 750 μL of 200 mM phosphate buffer/D_2_O, containing 1.4 mM TSP (3-(trimethylsilyl)propionic acid sodium salt) as internal standard. Finally, 700 μL of the solution was transferred into a 5 mm NMR tube. NMR analyses were carried out on a Jeol JNM-ECZ 600R operating at the proton frequency of 600.17 MHz and equipped with a Jeol 5 mm FG/RO DIGITAL AUTOTUNE probe. ^1^H NMR experiments were carried out by using the following parameters: 298 K, 256 scans, residual water signal suppression with a presaturation pulse, 7.7 s relaxation delay, 90° pulse of 8.3 μs, 64 k data points, and 9,000 Hz spectral width. ^1^H spectra were referenced to methyl group signals of TSP (*δ*_H_ = 0.00 ppm) in D_2_O. Homonuclear ^1^H-^1^H TOCSY experiment was acquired with 96 scans, 8 k data points in *f*2 and 128 in *f*1, 50 ms mixing time, 2 s relaxation delay, and 9,000 Hz spectral width in both dimensions. Heteronuclear ^1^H-^13^C HSQC experiment was acquired with 88 scans, 8 k data points in *f*2 and 256 in *f*1, 3 s relaxation delay, and a spectral width of 9,000 Hz and 33,000 Hz for *f*2 and *f*1, respectively. ^1^H-^13^C HMBC experiment was acquired with 64 scans, 8 k data points in *f*2 and 256 in *f*1, 2.8 s relaxation delay, and a spectral width of 9,000 Hz and 39,000 Hz for *f*2 and *f*1, respectively. Spectra processing and signal integration were carried out with the JEOL Delta software (v5.3.1). For each metabolite, a characteristic signal was considered and integrated, normalizing the area to those of the methyl group signals of TSP, set to 100. Results were expressed as μg/mL of sample.

### Colorimetric analysis

The samples were analyzed at the initial time (t^0^) for their color character, with a colorimeter X-Rite MetaVue™ (X-Rite, Prato, Italy), equipped with full spectrum LED illuminant and an observer angle of 45°/0° imaging spectrophotometer. Samples that were stored in a refrigerator (4°C) were analyzed again in the same conditions after 2 (t^2w^) and 4 weeks (t^4w^). Cylindrical coordinates C*_ab_ and h_ab_ and the color distances (ΔE) were calculated according to the reference samples at t° sample, as known by the literature (Cairone et al., 2020).

## Results

### Determination of the minimum biofilm eradication concentration

**Figure 2:**
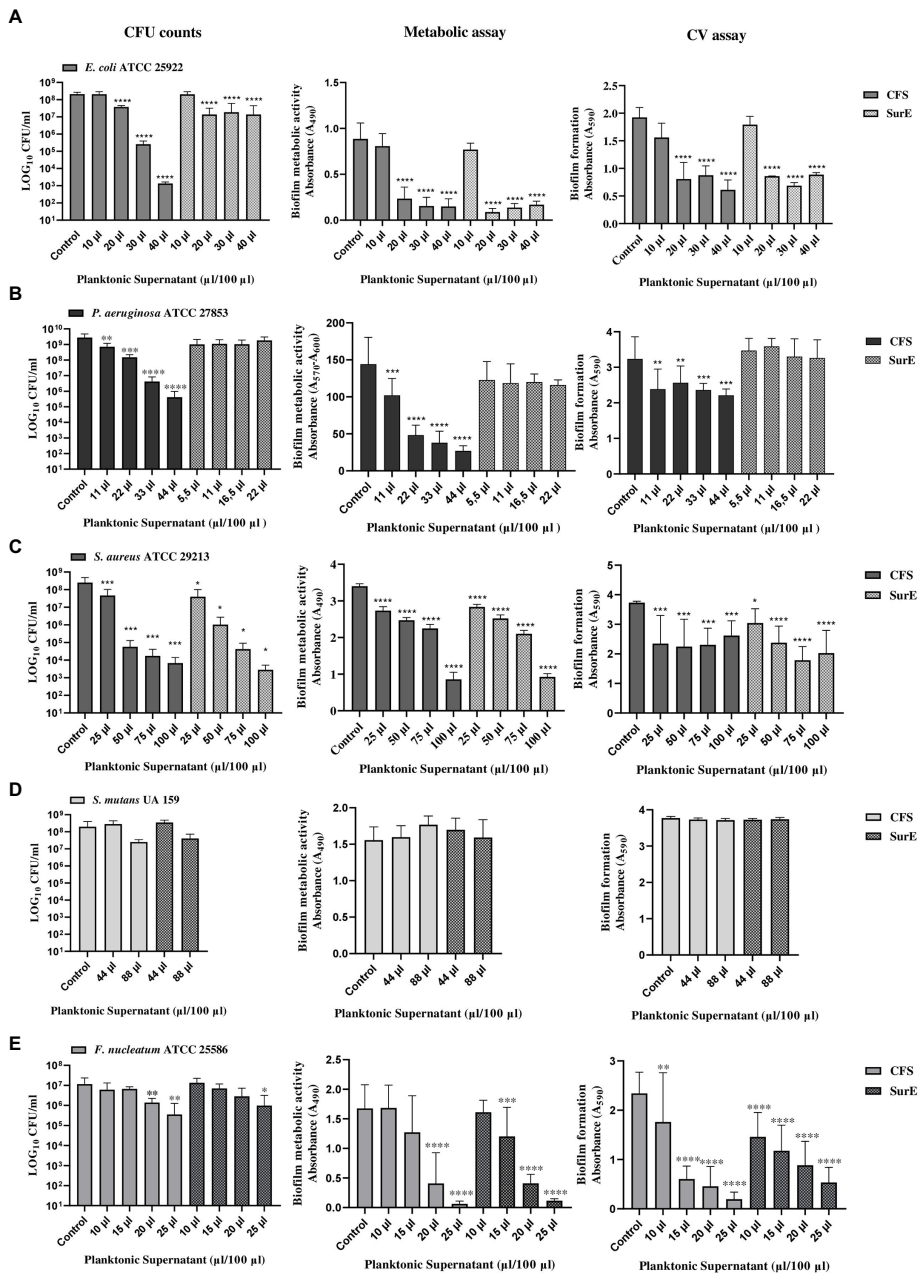
Determination of MBEC of CFS and SurE 10 K through CFU counts, metabolic assay, and CV assay vs. *Escherichia coli* ATCC 25922 **(A)**, *Pseudomonas aeruginosa* ATCC 27853 (metabolic assay) **(B)**, *Staphylococcus aureus* ATCC 29213 **(C)**, *Streptococcus mutans* UA 159 **(D)**, and *Fusobacterium nucleatum* ATCC25586 **(E)**. Data are presented as the mean of three replicates from two independent experiments. The statistical comparison between the control and treated samples was determined with a one-way ANOVA. The control was composed of media with the supplement of MRS broth. The error bars represent the standard deviation. The asterisks stand for *p*-value: *p < 0.05, **p < 0.01, ***p < 0.001, and ****p < 0.0001.

**Figure 3:**
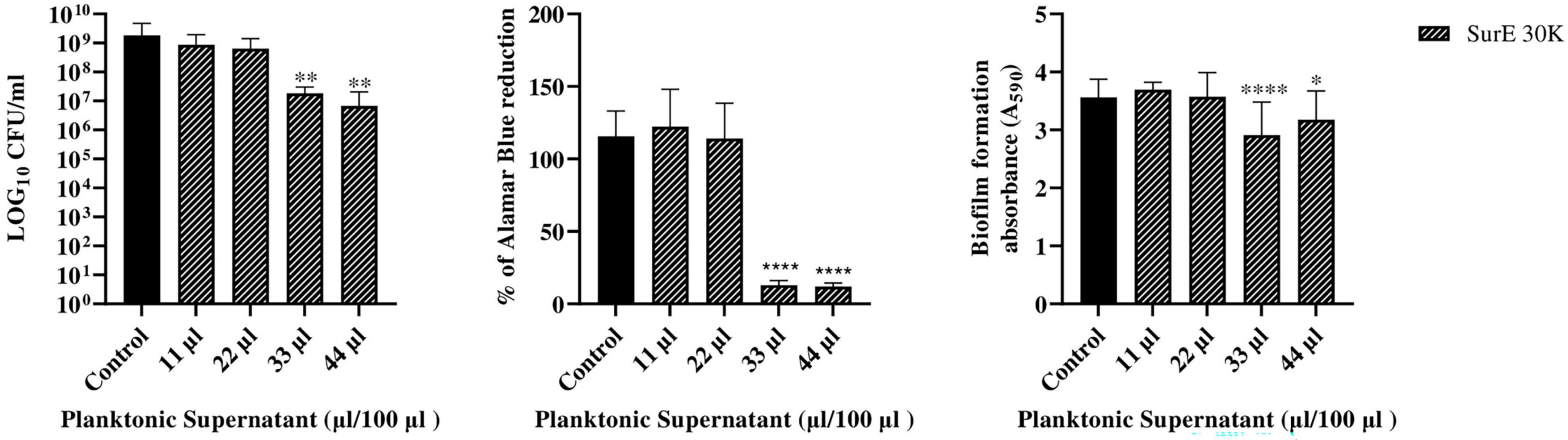
Determination of MBEC of SurE 30 K through CFU counts, metabolic assay, and CV assay vs. *P. aeruginosa* ATCC 27853. Data are presented as the mean of three replicates from two independent experiments. The statistical comparison between the control and treated samples was determined with a one-way ANOVA. The control was made up of media with the supplement of MRS broth. The error bars represent the standard deviation. The asterisks stand for *p*-value: *p < 0.05, **p < 0.01, and ****p < 0.0001.

The ability of factors secreted by *L. reuteri* DSM 17938 to eradicate biofilms produced by different pathogenic bacteria was evaluated by using three different methods to obtain more reliable results. Two independent experiments, performed in triplicate, have been carried out for each method. The methods used were: CFU counting, biofilm metabolic activity, and crystal violet (CV) assay, followed by fluorescent microscopy qualitative analysis (data not shown), which represented an additional confirmation. The exact correspondence of the results obtained guarantees us the accuracy and reproducibility of the data. The CFS showed the capability to eradicate pre-formed biofilms developed by *E. coli* ATCC 25922, *P. aeruginosa* ATCC 27853, *S. aureus* ATCC 29213, and *F. nucleatum* ATCC 25585 (Figure 2). In particular, the effect of the CFS at 2 × MIC (corresponding to 20 μL/100 μL) on the eradication of *E. coli* pre-formed biofilm was confirmed by the significant reduction in CFU counts and metabolic activity, as well as a statistically significant reduction in the biofilm biomass, as shown in Figure 2A. While the effect of CFS on the eradication of *P. aeruginosa* and *S. aureus* mature biofilms was detected at 2 × MIC or at MIC (corresponding to 11 μL/100 μL and 25 μL/100 μL, respectively; Figures 2B,C). On the contrary, CFS was not capable of eradicating the biofilm developed by *S. mutans* UA 159 (Figure 2C). Regarding the SurE 10 K, it showed a value of MBEC comparable to those of CFS vs. *E. coli* and *S. aureus* (see Figures 2A,C). On the contrary, it did not work against *P. aeruginosa*, suggesting that the molecules responsible for the inhibitory activity are contained in the CFS fraction (Figure 2B). To demonstrate this, we performed an intermediate fractionation by using 30 K columns. The results obtained demonstrated that SurE 30 K is capable of eradicating *P. aeruginosa* mature biofilm at 3 × MIC (corresponding to 33 μL/100 μL; Figure 3). Finally, SurE 10 K, as previously demonstrated for CFS, did not eradicate *S. mutans* biofilm (Figure 2D). Regarding the antibiofilm activity of the CFS against the *F. nucleatum* ATCC 27853 biofilm, the results showed a statistical reduction at 4 × MIC for CFS and 5 × MIC for SurE 10 K, demonstrating a more powerful eradication ability of the CFS.

### SurE 30 K MIC and MBEC vs. *Pseudomonas aeruginosa* ATCC 27853

Since SurE 10 K did not show any antibiofilm effect toward *P. aeruginosa*, it was decided to test SurE ultrafiltrated through a 30 kDa (SurE 30 K) filter to check if the 10 kDa filtration did not allow the passage into the eluted sample of the compound(s) responsible for the antibiofilm activity. SurE 30 K showed a MIC of 11 μL/100 μL toward *P. aeruginosa*, the same as in CFS. The MBEC value corresponded to 33 μL/100 μL (3 × MIC; Figure 3).

### Determination of the minimum biofilm inhibitory concentration

We next evaluated the ability of the secreted factors to inhibit biofilm formation. In particular, the capability of the CFS and SurE 10 K to inhibit the biofilm formation of the species previously mentioned was determined by the evaluation of the Minimum Biofilm Inhibitory Concentration (MBIC) by using, as previously mentioned, the CFU counting, biofilm metabolic activity, and CV assay. Both the CFS and SurE 10 K were not capable of inhibiting biofilm formation in any of the microorganisms tested. No significant reduction in the number of CFUs, biofilm metabolic activity, or biomass of the biofilm was detected (Supplementary Figure S1).

### Minimum inhibitory concentration of CFS and SurE 10 K vs. *Lacticaseibacillus rhamnosus* ATCC 53103*, Lacticaseibacillus paracasei* CNCM I-1572, and *Lactobacillus acidophilus* LA14 ATCC SD 5212

We also tested the potential antimicrobial activity of CFS and Sur E 10 K vs. other probiotic strains such as *L. rhamnosus* ATCC 53103, *L. paracasei* CNCM I-1572, and *L. acidophilus* LA14ATCC SD 5212 to evaluate the selective toxicity of these postbiotics against pathogenic bacteria with respect to probiotic strains. CFS and SurE 10 K did not show any inhibitory effect toward *L. rhamnosus* and *L. paracasei.* Regarding *L. acidophilus*, the MIC was 88 μL/100 μL for both CFS and SurE 10 K (Table 1).

**Table 1:** Evaluation of the MIC of CFS and SurE 10 K, obtained by *L. reuteri* DSM 17938, toward three probiotic strains.

Bacterial strains	CFS MIC (μL/100 μL)	SurE 10 K MIC (μL/100 μL)
*L. rhamnosus* ATCC 53103	**>**88	**>**88
*L. paracasei* CNCM I-1572	**>**88	**>**88
*L. acidophilus LA14* ATCC SD 5212	88	88

### NMR-based metabolomic profile

The NMR analysis of the considered samples (including MRS broth as the blank) allows identifying two organic acids (lactate and formate), nine amino acids (alanine, valine, glycinebetaine, isoleucine, leucine, glycine, phenylalanine, tyrosine, and tryptophan), and choline by means of 2D experiments and literature data (Di Matteo et al., 2021; Spano et al., 2021) as reported in Supplementary Table S1 and Supplementary Figures S2–S6. Comparing the NMR results obtained in Table 2, lactate and formate were identified among organic acids, whereas leucine, isoleucine, valine, alanine, glycine, tyrosine, phenylalanine, tyrosine, and tryptophan were revealed among amino acids. Choline and glycinebetaine were also present. Formate and glycine were detected in CFS samples only.

**Table 2:** Quantitative results obtained from the NMR analysis of samples subtracted from their blank (MRSB).

Metabolite	CFS	SurE 10 K
Lactate	6746.00	8427.49
Formate	28.49	–
Leucine	236.04	308.93
Isoleucine	92.08	114.03
Valine	158.56	213.97
Alanine	380.62	523.70
Glycinebetaine	63.33	75.71
Glycine	550.09	–
Tyrosine	29.19	38.09
Phenylalanine	113.28	151.04
Tryptophan	3.20	8.38
Choline	10.72	15.55

Results are expressed as μg/ml of sample. “–” means “not detected.”

### Determination of the MIC of lactic acid vs. *Staphylococcus aureus*

**Figure 4:**
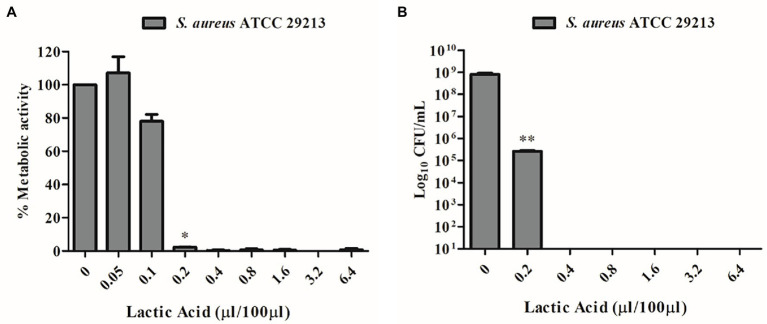
**(A)** MIC determination of lactic acid vs. *S. aureus* ATCC 29213 using the XTT assay. **(B)** MBC determination of lactic acid vs. *S. aureus* ATCC 29213 using the CFU count.

Due to the well-known antimicrobial activity of lactic acid-producing bacteria and the high content of this organic acid detected by quantitative NMR analyses (Table 2), lactic acid MIC was detected by unaided eye at 0.2 μL/100 μL and was also confirmed by XTT assay (Figure 4A). The minimum bactericidal concentration of lactic acid was determined at 0.4 μL/100 μL through the CFU count (Figure 4B).

### Stability studies using reflectance colorimetry

**Figure 5:**
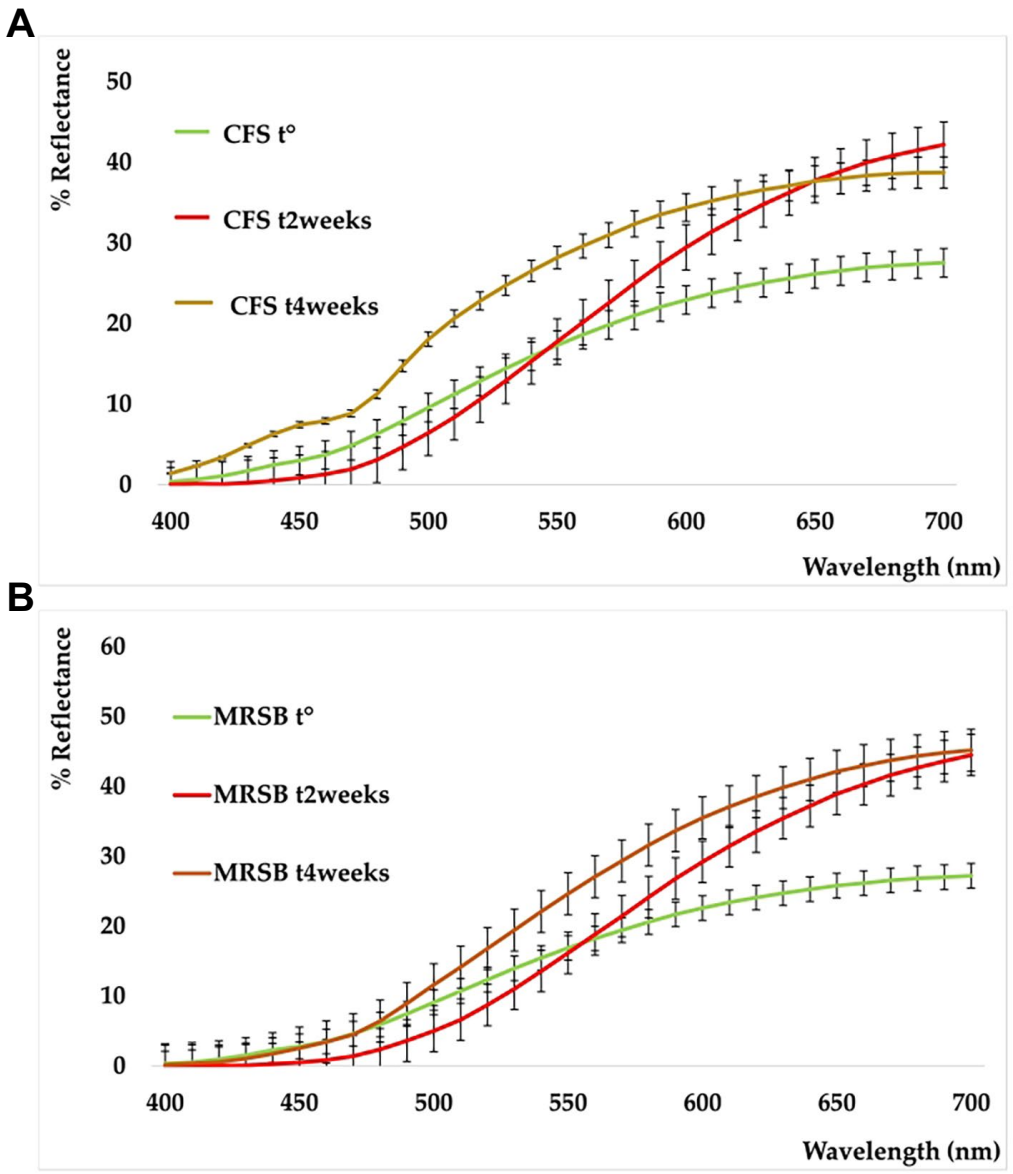
Reflectance curves of CFS **(A)** and planktonic supernatant blank (MRSB) **(B)**, at t^0^ (green), t^2weeks^ (red), and t^4weeks^ (ocher).

The shelf-life of the samples was monitored after 2 and 4 weeks of storage at 4°C. All the detected color parameters are reported in Supplementary Table S2. Data showed the color differences existing among samples, both in terms of elapsed time and different analyzed samples. This is also shown by the sample reflectance curves reported in Figure 5. In the CFS (A), the variation with respect to t° is particularly marked at t^2w^ and assumes the mean of a powerful whitening. This trend, however, is well delineated also in the medium used as reference (MRSB, B) at t^2w^, so that the difference between the two systems remains low (ΔE = 4.87, CFS t^2w^ vs. MRSB t^2w^). On the contrary, an evident change is denoted after 4 weeks because the color of the supernatant is turning toward blue and green rather than increasing in red and yellow as in the medium (ΔE = 18.56 CFS t^4w^ vs. MRSB t^4w^).

## Discussion

Probiotics are considered an effective approach for treating infections and inhibiting the spread of antibiotic resistance. In particular, lactobacilli are involved in the process of biofilm formation on oral surfaces, exerting anti-adhesive activity against different pathogens given that they interfere with adhesion and microbial cells’ co-aggregation *via* the secretion of antagonistic substances such as bacteriocins, organic acids, enzymes, and biosurfactants (Söderling et al., 2011; Barzegari et al., 2020).

Different articles have shown that probiotics like *Limosilactobacillus fermentum* and *Lactiplantibacillus plantarum* and some of their derivatives possess an antibiofilm effect on *P. aeruginosa* (Sharma et al., 2018; Shokri et al., 2018). In particular, Sharma et al. demonstrated that postbiotics, produced by *L. fermentum*, can reduce quorum sensing signals needed for biofilm formation, confirming results from previous studies (Kanmani et al., 2011; Bulgasem et al., 2015; Sharma et al., 2018). Here, we demonstrated that CFS from *L. reuteri* can eradicate *P. aeruginosa* biofilm at 1 × MIC, even if its 10 kDa filtered fraction is not able to exert any eradication activity, suggesting that the molecules/compounds responsible for its action, have a molecular weight higher than 10 kDa. As previously mentioned, the antimicrobial activity of CFS is probably due to the synergistic effect of different molecules/compounds (Maccelli et al., 2020). These data confirm our hypothesis, according to which, in this case, the antibiofilm activity is associated with several compounds, some of them with a size higher than 30 kDa, potentially involved either in the disintegration or in the permeabilization of the biofilm EPS matrix, facilitating the penetration of other components with antimicrobial activity. Regarding this hypothesis, Sharma et al. proposed that some bacteriocins from different probiotics are able to create pores through the *P. aeruginosa* cell membrane, leading to a change in cell membrane integrity and inducing cell death; similarly, some bacteriocins produced by *Streptococcus pyogenes* FF22 are able to disrupt the membrane potential of a *S. aureus* biofilm by forming short-lived pores that allow the passage of ions, leading to a major ATP efflux from biofilms which is correlated with the antimicrobial activity of bacteriocins (Okuda et al., 2013; Sharma et al., 2018). Tunçer et al. also showed very interesting results related to the action of CFS from *S. salivarius* M18. The authors demonstrated that CFS increases the lipid unsaturation index of *P. aeruginosa* and decreases the content of cellular polysaccharides, factors that have a crucial role in the formation of *P. aeruginosa* biofilm (Tunçer and Karaçam, 2020).

Ghane et al. showed that CFS produced from different probiotics, in particular, *Lactobacillus rhamnosus* and *Lactobacillus paracasei*, had a strong uropathogenic *E. coli* (UPEC) biofilm inhibitory effect (Ghane et al., 2020). On the contrary, Al-Dhabi et al. showed that *L. reuteri* LR12 has an inhibition rate of 53% on *E. coli* biofilm, a 68% inhibition rate against *P. aeruginosa* biofilm, and a 100% inhibition rate vs. *S. aureus* biofilm (Al-Dhabi et al., 2020). These data support our results, although the efficacy is known to be strain-specific. In fact, we demonstrated a more powerful eradication by *L. reuteri* DSM 17938 CFS against *S. aureus* and *P. aeruginosa* biofilms (1 × MIC), with respect to *E. coli*, where the MBEC value was higher (2× MIC). In contrast to our findings, Melo et al. showed that *L. fermentum* TCUESC01 can reduce the formation of *S. aureus* biofilm under sub-inhibitory concentrations, at 50% of the MIC, confirming that the inhibition growth ability of probiotics differs among strains (Keller et al., 2011; Melo et al., 2016).

Concerning *S. mutans*, our findings showed both that the MIC value is higher (44 μL/100 μL) when compared to other bacterial strains, including *F. nucleatum* (5 μL/100 μL), and that CFS does not have any biofilm eradication action until 2 × MIC concentration. It is important to take into consideration that the biofilm was developed in BHI supplemented with 1% of sucrose and that *S. mutans* uses sucrose from dietary sources for the synthesis of EPS, which act as a scaffold and contribute to its pathogenicity and antimicrobial resistance (Yang et al., 2019). We never supplemented MRSB media with glycerol, which is required by *L. reuteri* to produce reuterin (Maccelli et al., 2020). When considering the action of reuterin, it is important to consider that the amount of glycerol in the plaque is very low, therefore it may not be so important for oral health (Söderling et al., 2011). Yang et al. demonstrated that *L. reuteri* AN417 CFS is able to reduce *S. mutans* biofilm formation in the absence of reuterin. The authors developed *S. mutans* biofilm in the absence of sucrose, which was capable of increasing its pathogenicity, and water-insoluble glucan, derived from sucrose metabolism, and involved in *S. mutans* biofilm formation (Hasibul et al., 2018; Yang et al., 2021). The results in the literature are controversial, but it is known that considerable genotypic and phenotypic differences exist between *S. mutans* strains (Senpuku et al., 2018).

Besides reuterin, *L. reuteri* strains can produce different antimicrobial substances, including lactic acid, acetic acid, and ethanol. Data have evidenced the importance of the acidic environment in the interactions between *L. reuteri* and other microorganisms, in particular with the effect of lactic acid (Geraldo et al., 2020). To ensure that lactic acid was not the only player responsible for the antimicrobial activity despite its high content in both *L. reuteri* CFS and SurE 10 K, we evaluated the MIC of lactic acid against *S. aureus*. The results showed a MIC value of 0.2 μL/100 μL. Conversely, the MIC of *L. reuteri* CFS was 25 μL/100 μL, demonstrating that the inhibitory activity was not associated with the action of lactic acid alone but was derived from a synergistic effect among different molecules or compounds present in the supernatant and characterized by a molecular size lower than 10 kDa. In fact, 0.025 mL of CFS or SurE 10 K should contain 0.16 mg and 0.21 mg of lactic acid, respectively, based on the amounts reported in Table 2, whereas 0.2 μL of lactic acid corresponds to 0.24 mg (lactic acid density = 1,209 mg/mL). It is well-known that probiotics can produce some bacteriocin-like compounds that have been claimed to be more active at acid pH than at neutral or alkaline pH (Li et al., 2015); therefore, we could also hypothesize the presence of such compounds in CFS. Further studies are needed to better understand which molecules could contribute to the efficacy of CFS.

With regards to *F. nucleatum*, some articles in the literature have shown that some probiotics like *Bifidobacterium lactis* and *Bifidobacterium infantis* are able to cause an antagonistic effect toward periodontopathogens like *F. nucleatum* (Valdez et al., 2021). *Fusobacterium nucleatum* has the ability to co-aggregate with other bacteria, and this may be due to its long rod morphology that acts as a bridge in dental plaque (Kolenbrander and London, 1993). Moreover, *F. nucleatum* is able to reduce oxygen levels, contributing to the growth of less aerotolerant and more pathogenic bacterial species like *Porphyromonas gingivalis* (Bradshaw and Marsh, 1998; Llama-Palacios et al., 2020).

Many studies have demonstrated the efficacy of CFS and probiotic cells on *F. nucleatum* growth. Yang et al., for example, showed that the CFS produced by *L. reuteri* AN 417 was very effective against *P. gingivalis* growth but less effective on *F. nucleatum* and *S. mutans* (Yang et al., 2021). With regards to the antibiofilm activity toward *F. nucleatum*, a limited number of details are reported. Jiang et al. proved that *S. mutans* and *F. nucleatum* have a poorer ability to grow in mono-species biofilms and they demonstrated better abilities of adhesion and reproduction in dual-and/or multispecies biofilms (Jiang et al., 2016). In multi-species biofilms, neighbors can take advantage of the productivity of the community, in particular in the oral cavity, in which they develop dental plaque (Elias and Banin, 2012). In the end, the evaluation of the effect of *L. reuteri* DSM 17938 vs. other probiotic strains showed promising results given that the CFS did not demonstrate any inhibitory activity toward the probiotic strains tested but only toward pathogens. The results reported represent the first contribution to the evaluation of the antibiofilm activity of CFS produced by *L. reuteri* DMS 17938 vs. *F. nucleatum* mono-species biofilm. The capability of *L. reuteri* DSM 17938 CFS to inhibit *F. nucleatum* growth and eradicate its biofilm could represent a strategy to prevent the formation of polymicrobial dental plaque, avoiding the development of biofilm-related pathologies in the oral cavity. Further studies should be performed in order to determine the activity of CFS in multi-species biofilms, especially from species of clinical relevance that colonize the oral cavity, such as *S. mutans* and *P. gingivalis*.

The application of NMR spectroscopy for the metabolomic analysis of these matrices was effective for the identification and quantification of primary and secondary metabolites, giving important information with regards to the potential role of some of them in the observed anti-biofilm activity. Only one article regarding the NMR analysis of *Lactobacillus* vesicles has been found (Ñahui Palomino et al., 2019). In respect to the assignments present, a further identification was observed in this work by detecting citrate, malate, and glycinebetaine. The latter is an osmoprotectant metabolite that usually improves adaptation to environmental salt stresses, especially for those bacteria living in the mammalian gut (e.g., *C. difficile*), where salinity could hamper bacterial growth (Michel et al., 2022). This is the first report revealing the presence of this compound in probiotic species as well. Generally, Sur E 10 K was richer in terms of all detected metabolites, despite the fact that formate and glycine were present only in the CFS. Lactic acid was the most abundant metabolite. Among amino acids, we found the following substances in the order of decreasing amounts: glycine > alanine > leucine > valine > phenylalanine. All these compounds were also detected *via* untargeted metabolomic profiling by means of high-resolution fourier transform ion cyclotron resonance mass spectrometry (FT-ICR MS) coupled with electrospray ionization source (ESI; Maccelli et al., 2020), but without any knowledge of the content.

To assess the stability of CFS and Sur E 10 K, we performed a fast, affordable, and non-destructive analysis based on reflectance colorimetry. Besides a well-known role in the consumer’s compliance toward a product, color could play a main role in assessing differences and peculiarities among the commodity class, as well as give information related to stability and allow the evaluation of their shelf-life. As previously explained, this is of particular significance in the case of rapidly evolving biological systems. In fact, color is influenced and quickly modified by humidity, temperature, oxygen content, and metal presence, apart from many other factors correlated to the nature of the analyzed matrix, its solid or liquid state, the presence of enzymes, the molecular composition, and light exposure (Cesa et al., 2017).

To the best of our knowledge, the tristimulus colorimetric analysis was applied for the first time to evaluate the color characters, the differences, and the stability of CFS derived from *L. reuteri*. Only some articles are available in the literature which deal with the color onset in cyanobacteria forming biofilm (Sanmartín et al., 2010, 2012), while some other articles deal with the color evaluation of fermented foods in which lactobacilli were grown (Pelicano et al., 2003; Antunes et al., 2013; Zhu et al., 2020; Wang et al., 2021). In previous studies, the change of color in milk was evaluated in relation to the microorganism growth based on time and temperature (Ziyaina et al., 2019), and the ΔE scale, as proposed by Limbo and Piergiovanni (2006), was used to discriminate colors. The results confirmed that these natural matrices can be subjected to degradation phenomena after 2 and 4 weeks of storage and that they must be conserved at temperatures below 4°C to preserve the bioactive components.

The results indicated that CFS and its sub-fractions from *L. reuteri* DSM 17938 have a promising potential for the eradication of biofilms produced by clinically relevant species that are biofilm producers and responsible for nosocomial and oral infections. The potential of CFS should be thoroughly investigated in order to understand novel inhibitory compounds as new antibiofilm agents. The NMR metabolomic profile discriminated specific metabolites, which, along with MS data, can be considered the fingerprint of these natural matrices. Their stability tested spectrophotometrically for up to 4 weeks also gave new insights concerning storage time within the performance of biological experiments.

## Data availability statement

The original contributions presented in the study are included in the article/Supplementary material, further inquiries can be directed to the corresponding author.

## Author contributions

RG and SCa designed the project. RG and SCa designed the experiments, discussed the results, and drafted the manuscript. IV, VP, BM, MS, SCe, and FS performed the experiment consisting of the isolation of CFS, evaluation of the antibiofilm activity, and analysis of metabolomic/color profiles. RG, SCa, GG, and SR drafted the final editing of the manuscript and critically revised it. All authors contributed to the article and approved the submitted version.

## Funding

This study was supported by the company BioGaia AB, Stockholm Sweden, and the Ministero Italiano dell’Università e della Ricerca (MIUR) FAR2020 Grant, held by RG.

## Conflict of interest

SR and GG are currently employed by the company BioGaia AB. RG and SCa have received a research grant from Company BioGaia which partially supported the project.

The remaining authors declare that the research was conducted in the absence of any commercial or financial relationships that could be construed as a potential conflict of interest.

## Publisher’s note

All claims expressed in this article are solely those of the authors and do not necessarily represent those of their affiliated organizations, or those of the publisher, the editors and the reviewers. Any product that may be evaluated in this article, or claim that may be made by its manufacturer, is not guaranteed or endorsed by the publisher.
