# Supplementary material for: Antibiofilm activity and NMR-based metabolomic characterization of cell-free supernatant of Limosilactobacillus reuteri DSM 17938
Source: Front Microbiol. 2023 Feb 20;14:1128275. doi: 10.3389/fmicb.2023.1128275 (PMC9986594; doi:10.3389/fmicb.2023.1128275)
Supplement: Supplementary file 1 [file Data_Sheet_1.docx]

**SUPPLEMENTARY**

**Antibiofilm Activity and NMR-based Metabolomic Characterization of Cell Free Supernatant of *Limosilactobacillus reuteri* DSM 17938**

Irene Vitale^1ɸ^, Mattia Spano^2,ɸ^, Valentina Puca^1^, Simone Carradori^1^, Stefania Cesa^2^, Beatrice Marinacci^1^, Francesca Sisto^3^, Stefan Roos^4,5^, Gianfranco Grompone^5^, Rossella Grande^1,6,*^

^1^Department of Pharmacy, “G. d’Annunzio” University of Chieti-Pescara, 66100 Chieti, Italy.

^2^Department of Drug Chemistry and Technology, Sapienza University of Rome, Piazzale Aldo Moro 5, 00185 Rome, Italy.

^3^Department of Biomedical, Surgical and Dental Sciences, University of Milan, 20133 Milan, Italy.

^4^Department of Molecular Sciences, Swedish University of Agricultural Sciences, 750 07 Uppsala, Sweden.

^5^BioGaia, SE-103 64 Stockholm, Sweden.

^6^Center for Advanced Studies and Technology (CAST), “G. d’Annunzio” University of Chieti-Pescara, 66100 Chieti, Italy.

^ɸ^IV and MS equally contributed to the work.

*Corresponding author: Rossella Grande, Department of Pharmacy, “G. d’Annunzio” University of Chieti-Pescara, 66100 Chieti, Italy; rossella.grande@unich.it


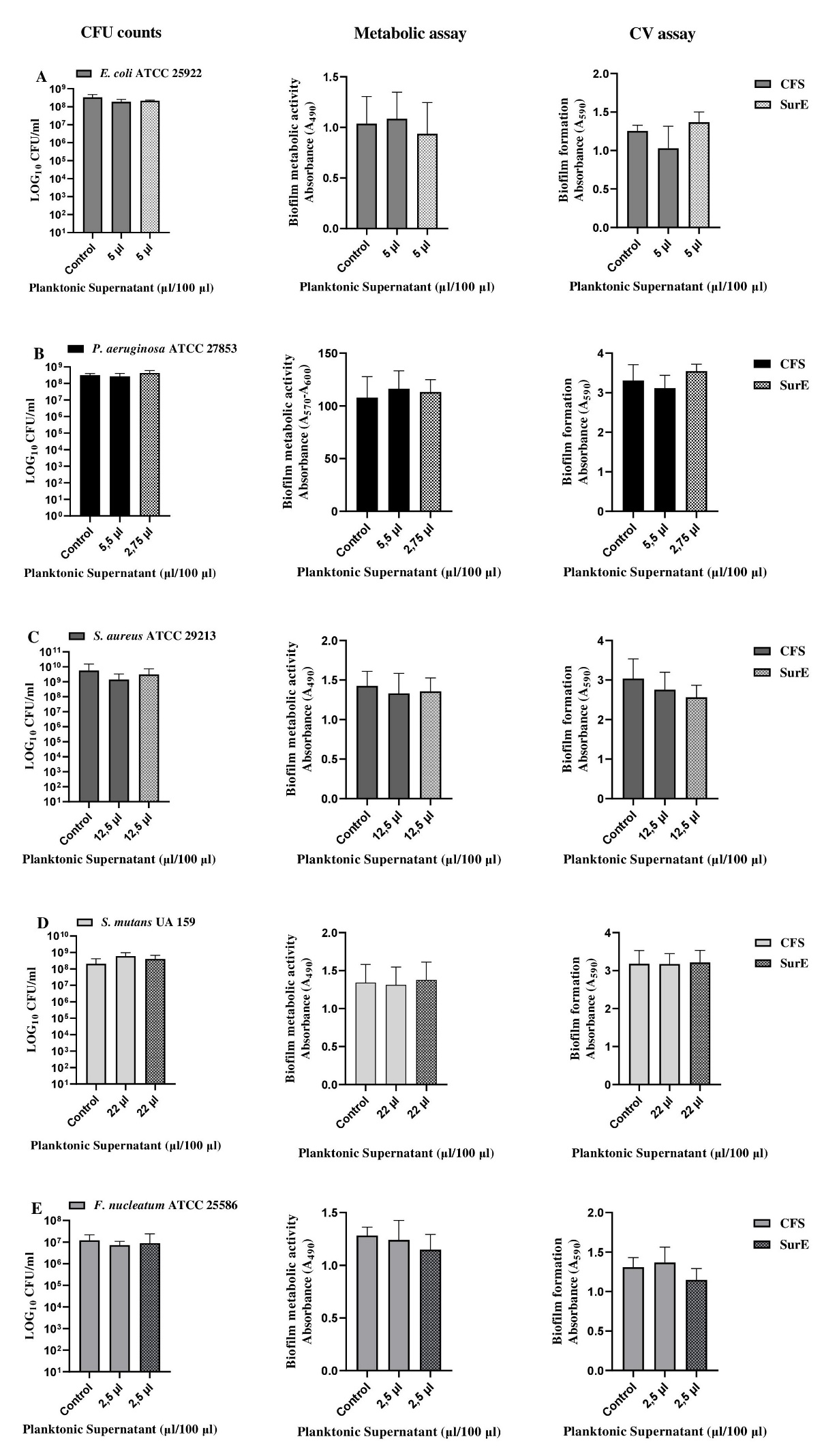


**Figure S1**. Determination of MBIC of CFS and SurE 10K through CFU counts, metabolic assay and CV assay versus *E. coli* ATCC 25922 (**A**), *P. aeruginosa* ATCC 27853 (**B**), *S. aureus* ATCC 29213 (**C**), *S. mutans* UA 159 (**D**) and *F. nucleatum* ATCC 25585 (**E**). Data are presented as the mean of three replicates of two independent experiments. The statistical comparison between control and treated samples was determined with one way ANOVA. The control was composed by media with the supplement of MRSB.


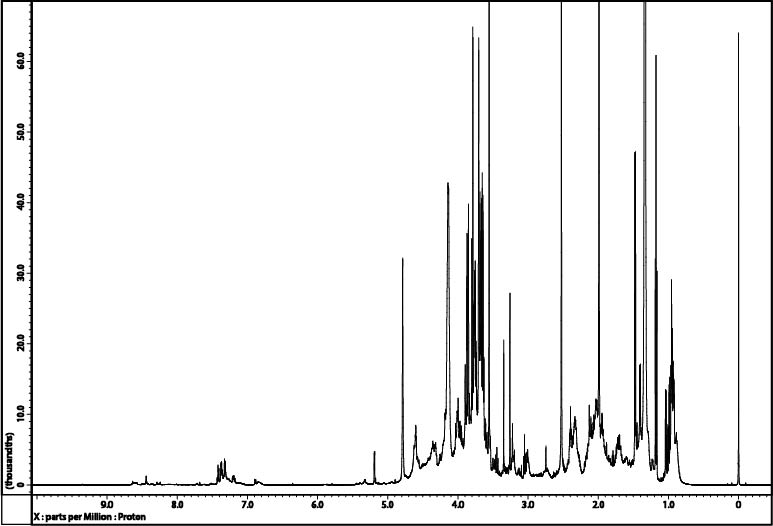


**Figure S2.** 600.17 MHz ^1^H NMR spectrum of CFS sample dissolved in 200 mM phosphate buffer/D_2_O containing TSP 1.4 mM.

**
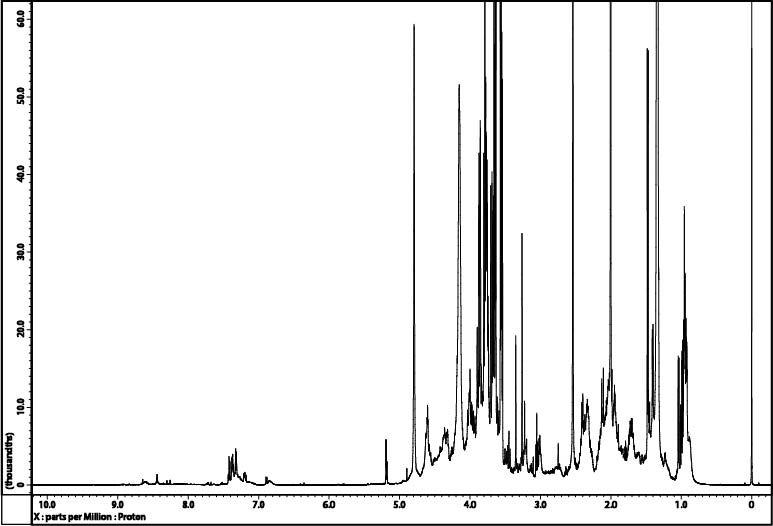
**

**Figure S3.** 600.17 MHz ^1^H NMR spectrum of SurE 10K sample dissolved in 200 mM phosphate buffer/D_2_O containing TSP 1.4 mM.


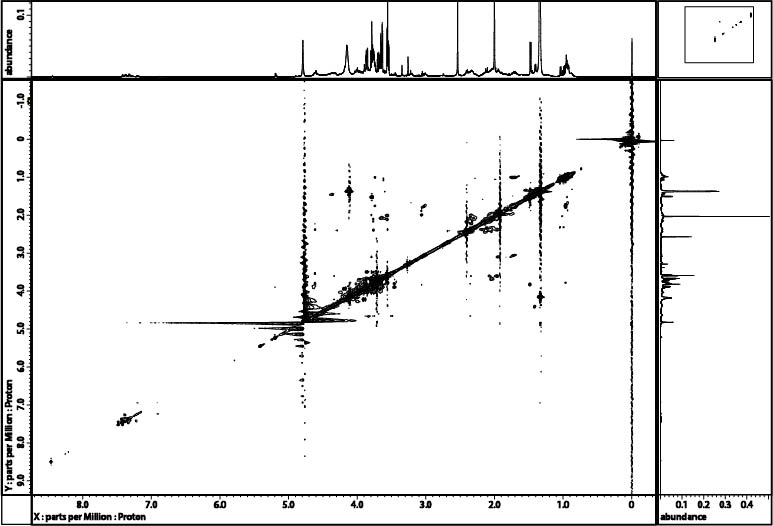


**Figure S4.** ^1^H-^1^H TOCSY spectrum of SurE 10K sample dissolved in 200 mM phosphate buffer/D_2_O containing TSP 1.4 mM.

**
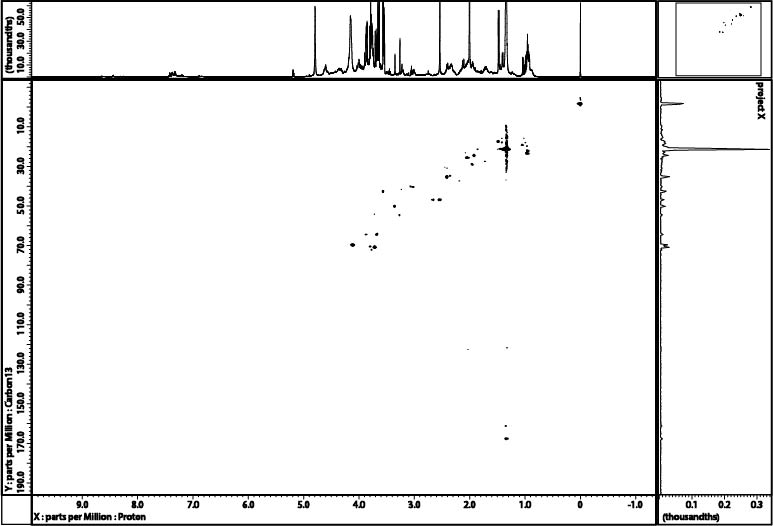
**

**Figure S5.** ^1^H-^13^C HSQC spectrum of SurE 10K sample dissolved in 200 mM phosphate buffer/D_2_O containing TSP 1.4 mM.


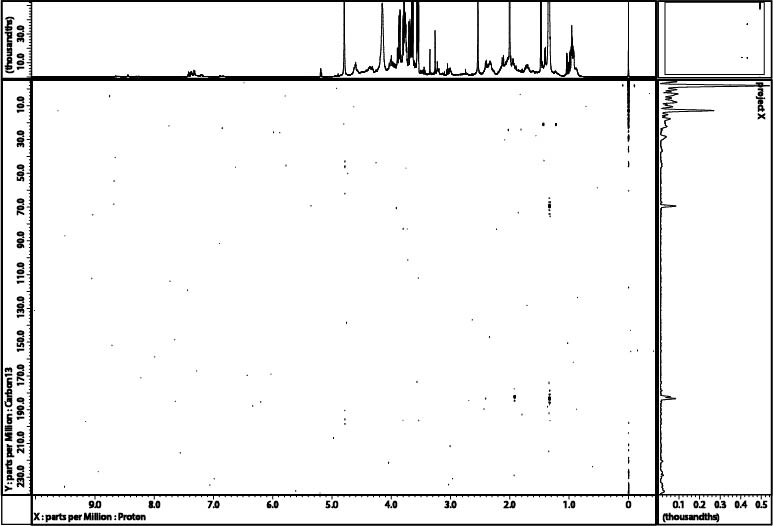


**Figure S6.** ^1^H-^13^C HMBC spectrum of SurE 10K sample dissolved in 200 mM phosphate buffer/D_2_O containing TSP 1.4 mM.

**Table S1.** Metabolites identified in the 600.17 MHz ^1^H NMR spectra of CFS and SurE 10K dissolved in 200 mM phosphate buffer/D_2_O containing TSP 1.4 mM. Asterisks (*) indicate signals selected for integration.

| **Metabolite** | **Assignment** | **^1^H (ppm)** | **Multiplicity [*J*(Hz)]** | **^13^C (ppm)** |
| --- | --- | --- | --- | --- |
|  |  |  |  |  |
| **Organic acids** |  |  |  |  |
|  |  |  |  |  |
| Lactate^a,b^ | β-CH_3_ | *1.33 | d [6.9] | 21.4 |
|  | α-CH | 4.12 | q [6.9] | 69.6 |
|  |  |  |  |  |
| Formate^a^ | HCOOH | *8.46 | s |  |
|  |  |  |  |  |
| **Amino acids** |  |  |  |  |
|  |  |  |  |  |
| Alanine^a,b^ | α-CH | 3.80 |  |  |
|  | β-CH_3_ | *1.49 | d [7.2] | 17.4 |
|  |  |  |  |  |
| Valine^a,b^ | β-CH | 2.28 |  | 30.5 |
|  | γ-CH_3_ | 1.00 | d [7.7] | 17.8 |
|  | γ’-CH_3_ | *1.05 | d [7.1] | 19.2 |
|  |  |  |  |  |
| Glycinebetaine^a,b^ | N(CH_3_)_3_+ | *3.27 | s | 55.1 |
|  |  |  |  |  |
| Isoleucine^a,b^ | β-CH | 1.99 |  |  |
|  | γ-CH_3_ | *1.01 | d [6.9] | 15.8 |
|  |  |  |  |  |
| Leucine^a,b^ | γ-CH | 1.74 |  |  |
|  | δ-CH_3_ | *0.97 | d [6.2] | 23.2 |
|  | δ’-CH_3_ | 0.96 | d [6.2] | 22.3 |
|  |  |  |  |  |
| Glycine^a^ | α-CH_2_ | *3.56 | s | 42.5 |
|  |  |  |  |  |
| Phenylalanine^a,b^ | CH-2,6 | 7.33 | m |  |
|  | CH-4 | 7.38 | m |  |
|  | CH-3,5 | *7.43 | m |  |
|  |  |  |  |  |
| Tyrosine^a,b^ | CH-3,5 | 7.20 | d [8.6] |  |
|  | CH-2,6 | *6.90 | d [8.6] |  |
|  |  |  |  |  |
| Tryptophan^a,b^ | CH-4 | *7.74 | d [7.9] |  |
|  | CH-7 | 7.55 | d [8.2] |  |
|  |  |  |  |  |
| **Miscellaneous metabolites** |  |  |  |  |
|  |  |  |  |  |
| Choline^a,b^ | ^+^N(CH_3_)_3_ | *3.21 | s |  |

^a^metabolite quantified in CFS; ^b^metabolite quantified in SurE 10K.

**Table S2.** Colorimetric CIEL*a*b* parameters of analyzed samples.

|  | **L*** | **a*** | **b*** | **C*** | **h°** | **ΔL*** | **Δa*** | **Δb*** | **ΔC*_ab_** | **Δh_ab_** | **ΔE** |
| --- | --- | --- | --- | --- | --- | --- | --- | --- | --- | --- | --- |
| **CFS t°** | 47.91 | 6.92 | 44.25 | 44.79 | 81.11 |  |  |  |  |  |  |
| **CFS t^2w^** | 49.74 | 17.88 | 65.16 | 67.57 | 74.65 | 1.84  Lighter | 10.96  More red | 20.91  More yellow | 22.78  Brighter | -6.20  More red | 23.68 |
| **CFS t^4w^** | 58.84 | 3.98 | 43.79 | 43.97 | 84.81 | 10.94  Lighter | -2.95  More green | -0.46  More blue | -0.82  Opaque | 2.87  More green | 11.34 |
|  | | | | | | | | | | | |
| **MRSB t°** | 47.37 | 7.36 | 44.76 | 45.36 | 80.66 |  |  |  |  |  |  |
| **MRSB t^2w^** | 48.44 | 21.43 | 68.23 | 71.51 | 72.56 | 1.06  Lighter | 14.07  More red | 23.47  More yellow | 26.16  Brighter | -8.05  More red | 27.39 |
| **MRSB t^4w^** | 56.23 | 12.17 | 60.24 | 61.45 | 78.58 | 8.86  Lighter | 4.81  More red | 15.48  More yellow | 16.10  Brighter | -1.92  More red | 18.47 |

Each reported value is the mean of four measurements. ΔE, calculated as: [(L*_2_–L*_1_)^2^ + (a*_2_–a*_1_)^2^ + (b*_2_–b*_1_)^2^]^1/2^, represents the overall color variation, respect to the corresponding samples at t°, used as references.
